# Novel Approaches to In-Situ ATR-FTIR Spectroscopy and Spectroscopic Imaging for Real-Time Simultaneous Monitoring Curing Reaction and Diffusion of the Curing Agent at Rubber Nanocomposite Surface

**DOI:** 10.3390/polym13172879

**Published:** 2021-08-27

**Authors:** Shun Muroga, Yu Takahashi, Yuta Hikima, Seisuke Ata, Sergei G. Kazarian, Masahiro Ohshima, Toshiya Okazaki, Kenji Hata

**Affiliations:** 1 CNT-Application Research Center, National Institute of Advanced Industrial Science and Technology, Tsukuba Central 5, 1-1-1, Higashi, Tsukuba 305-8565, Japan; ata-s@aist.go.jp (S.A.); toshi.okazaki@aist.go.jp (T.O.); kenji-hata@aist.go.jp (K.H.); 2 Department of Chemical Engineering, Graduate School of Engineering, Kyoto University, Kyoto 615-8510, Japan; takahashi.yu.76c@st.cheme.kyoto-u.ac.jp (Y.T.); ohshima.masahiro.2w@kyoto-u.ac.jp (M.O.); 3 Department of Chemical Engineering, Imperial College London, South Kensington Campus, London SW7 2AZ, UK

**Keywords:** in situ attenuated total reflection infrared spectroscopy, curing reaction, diffusion, triallyl isocyanurate, polymer nanocomposite, fluorine rubber, carbon nanotube, Fourier transform infrared spectroscopic imaging

## Abstract

Here, we propose a novel attenuated total reflection Fourier transform infrared (ATR-FTIR) spectroscopy method for simultaneously monitoring the curing reaction and the diffusion behavior of curing agents at the surface of rubber in real-time. The proposed scheme was demonstrated by fluorine rubber (FKM) and FKM/carbon nanotube (CNT) nanocomposites with a target curing agent of triallyl-isocyanurate (TAIC). The broadening and the evolution of the C=O stretching of TAIC were quantitatively analyzed to characterize the reaction and the diffusion. Changes in the width of the C=O stretching indicated the reaction rate at the surface was even faster than that of the bulk as measured by a curemeter. The diffusion coefficient of the curing agent in the course of heating was newly calculated by the initial increase in the absorbance and our model based on Fickian diffusion. The diffusion coefficients of TAIC during curing were evaluated, and its temperature and filler dependency were identified. Cross-sectional ATR-FTIR imaging and in situ ATR-FTIR imaging measurements supported the hypothesis of the unidirectional diffusion of the curing agent towards the heated surface. It was shown that our method of in situ ATR-FTIR can monitor the degrees of cure and the diffusion coefficients of curing agents simultaneously, which cannot be achieved by conventional methods, e.g., rheological measurements.

## 1. Introduction

Rubbers have become indispensable materials in various applications in our daily lives, such as sealing materials, tires, and stretchable devices. The advantages of rubbers in these industrial applications are their lightweight, soft/flexible, shock-resistant, and moldable characteristics. The elasticity is particularly important for any type of rubber application. The elasticity of rubber products is controlled by both the reinforcing fillers and the curing reaction of the polymer chain. For example, carbon black [1,2,3], silica [4,5], calcium carbide [6], halloysite [7], clay [8,9], carbon nanotube (CNT) [10,11,12,13,14,15,16,17], and graphene-based fillers [18,19,20,21] are used as the fillers to improve the mechanical properties of rubber materials. In the curing reaction, a three-dimensional network is formed with polymer chains and curing agents. Such a network largely affects the hardness, compressive force for sealing, and long-term stability of rubber products. Precise control of the curing behavior of rubber in the presence of fillers is important to obtain the desired properties with stable production.

To control the curing behaviors of rubbers, monitoring and evaluating the curing behaviors play an important role in obtaining a deeper understanding of rubber products. Curemeter, which detects the elasticity of rubber samples during heating, are widely used to monitor curing behavior through rheological properties. An increase in elastic torque synchronized with an oscillating force is related to the progression of the curing reaction. The elastic torque is normalized by the minimum and maximum elastic torque values and used as an indicator of the degree of cure to investigate the curing kinetics. Differential scanning calorimetry (DSC) is also used to evaluate the curing reactions of rubbers from the time evolution of exothermal enthalpy. Isothermal DSC curves are analyzed in a similar manner to the curing curves of the curemeter. For example, non-isothermal heating curves are analyzed using the Kissinger method [22] or the Ozawa method [23] to evaluate the curing kinetics. These methods of rheological measurement and thermal analysis are quite effective for characterizing the curing behavior of rubbers in bulk.

In practice, rubber/filler compounds contact the mold surface during processing, such as compression molding, transfer molding, and injection molding. A number of studies have been conducted on the precise control of curing behavior in the processing. Numerical simulations with the kinetic curing reaction models were performed to seek the optimum curing conditions of thick rubbery parts [24,25]. These simulations focused mostly on the inhomogeneity of thermal conduction in the thick rubber parts. Since uncured agents diffuse toward the surface of rubber, which contaminates the mold and products [26], evaluations of not only the curing reaction but also the mass transfer of curing agents are essential for a deeper understanding of the curing process of rubbery products. The conventional methods of using the curemeter or the DSC were not good enough to detect the mass transfer of curing agent during the curing process. Diffusion behaviors in thermoplastics are generally characterized by the weight of chemical species diffused over the surface [27]. However, a simultaneous characterization of the reaction and diffusion during the curing process is quite difficult. Therefore, a new method of analyzing the reaction and diffusion of the curing agent simultaneously is required to obtain accurate information on the kinetics of rubbers at the mold surface. If the reaction and diffusion behavior of rubber at the mold surface can be simultaneously evaluated, the information on the kinetics is useful to determine proper molding conditions and compositions of rubber products, including curing agents and fillers.

In this study, we applied vibrational spectroscopic techniques to develop a real-time monitoring method for rubber nanocomposites. Attenuated total reflection Fourier transform infrared (ATR-FTIR) spectroscopy [28] was particularly effective because of its chemical specificity, surface sensitivity (penetration of evanescent waves), and applicability to the rubber nanocomposites that contain fillers with high absorption (especially carbon fillers). We selected triallyl isocyanurate (TAIC) as a target curing agent to monitor and used fluorine rubber (FKM) as a model matrix. TAIC is widely used as a trifunctional curing agent for radical curing (heat or electron beam) of plastics and rubbers for toughening and improving thermal stability [29,30,31,32,33]. Our previous work reported that the broadening of the peak of C=O in triallyl isocyanurate rather than that of cleaved C=C was highly correlated with the degree of cure due to the steric hindrance induced by the curing reaction [34]. The changes in the C=O by steric hindrance was quantitatively validated by the relationship between the crosslinking density and the Young’s modulus and the robustness of the approach against curing temperature, curing time, filler kind, and filler content was clearly demonstrated. This approach of characterizing the curing reaction from C=O has potential since the phenomenon of restricting the vibration of C=O from the reduced free volume through network formation by curing is not unique to fluorine rubber. In the present study, we further extended the investigation to develop a method of in situ measurement by heating ATR crystals and acquiring data of time-series spectra to simulate a situation of heating rubber surface during a molding process. In this article, in situ ATR-FTIR spectroscopy and spectroscopic imaging are used to characterize the time-evolution of the curing behavior for kinetic analysis during heating. To see the effects of the filler, the CNT was chosen as the filler because CNT is one of the fillers widely used for the dramatic improvement of mechanical properties and thermal stability; however, its radical trapping effect may affect the curing reaction in some cases. To investigate the applicability of our proposed method for characterizing reaction and diffusion behaviors at the rubber surface using in situ ATR-FTIR spectroscopy, the effects of the curing temperatures and the filler on the behaviors during heating were identified from evaluating FKM and FKM/CNT nanocomposite.

## 2. Materials and Methods

FKM samples were composed of tetrafluoroethylene hexafluoropropylene vinylidene fluoride (Dai-el G-912, Daikin Co., Ltd., Osaka, Japan), 4.0 per hundred rubber (phr) of triallyl isocyanurate (TAIC, Nippon Kasei Chemical Co., Ltd., Tokyo, Japan) as a curing agent and 1.5 phr of 2,5-dimethyl-2,5-di(*t*-butyl peroxy)hexane (Perhexa 25B, NOF Corporation, Tokyo, Japan) as a curing initiator. The chemical structures of the materials are described in Figure S1. The single-walled carbon nanotube, which was synthesized by the super-growth method [35] (Zeonano, Zeon Co., Ltd., Tokyo, Japan), was used to fabricate the FKM/CNT samples. FKM and FKM/CNT samples were hot-pressed into 1-mm-thick pieces using a small hot press machine (AS ONE Co., Ltd., Osaka, Japan) at 20 MPa mechanical pressure and a temperature of 80 °C. The temperature of the hot-pressing was low enough to prevent the progress of the curing reaction during the hot-pressing.

**Figure 1:**
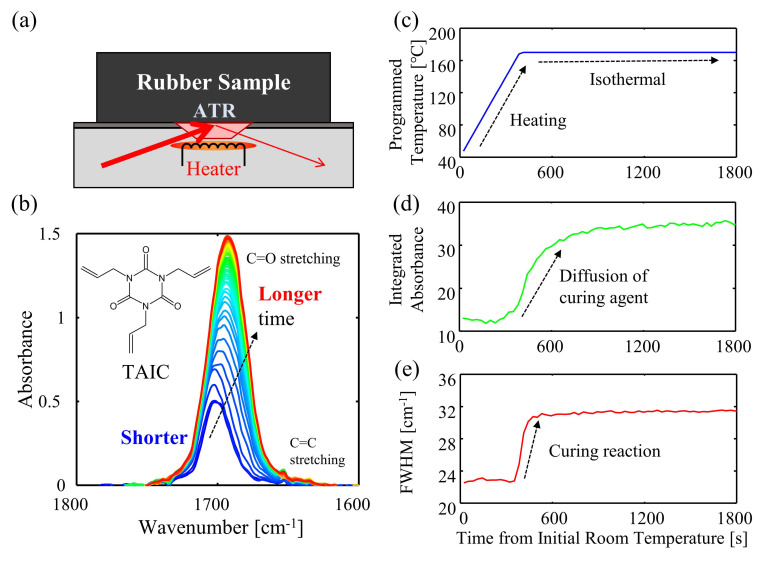
(**a**) Schematics of in situ ATR-FTIR spectroscopy to monitor rubber. (**b**) ATR-FTIR spectra of TAIC during heating. (**c**–**e**) Time series changes in the spectra: (**c**) programmed temperature, (**d**) integrated absorbance, and (**e**) FWHM of the C=O stretching band.

ATR-FTIR spectra were acquired using a Fourier transform infrared spectrometer (Spectrum 100, PerkinElmer, Inc., Massachusetts, USA) with an ATR accessory (Golden Gate, Specac, Inc., Orpington, UK) and a heating stage controlling the temperature of the ATR crystal (Figure 1a). Spectrum in a wavenumber range from 600 to 4000 cm^−1^ with spectral resolution of 4 cm^−1^ and a signal-averaging of four scans was successively recorded in 30-s intervals. The heating rate from room temperature to the designated heating temperature was set to 20 °C min^−1^ for all conditions. The measurements were conducted at four heating temperatures, 150, 160, 170, and 180 °C, to investigate the effect of heating temperature, and performed three times at each temperature. Each ATR-FTIR spectrum was subjected to the baseline correction and subsequently normalized by the absorbance of C–F stretching in FKM at 1396 cm^−1^, whose intensity was not changed during the curing process. The change in the TAIC concentration can be evaluated by the integrated absorbance of the C=O stretching band using Simpson’s rule in a similar manner of our previous work [34]. The penetration depth of evanescent waves is approximately 0.93 μm at 1700 cm^−1^ (C=O stretching region), and the refractive index is 2.42 (fluorine rubber matrix), which is sufficiently smaller than the sample thickness.

In our previous work, the C=O stretching of TAIC was related to the degree of cure in FKM prepared by different molding conditions regardless of fillers [34]. Here, we determined the degree of cure at the surface of rubber measured by in situ ATR-FTIR, $\alpha_{\mathrm{surf}}$, as follows:

(1) $$\alpha_{\mathrm{surf}}\left(t\right)=\frac{\Delta \nu \left(t\right)-{\Delta \nu }_{\min}}{\Delta \nu_{\max}-\Delta \nu_{\min}}$$

where Δν is the full width at half maximum (FWHM) of C=O stretching at each time t, $\Delta \nu_{\max}$ is the maximum FWHM of C=O stretching, and $\Delta \nu_{\min}$ is the minimum FWHM of C=O stretching.

To see the distributions of TAIC in the samples, ATR-FTIR spectroscopic imaging measurements were also carried out using a custom-made equipment in Kazarian’s group as recently reported [36,37,38]. The setup of the measurements consists of a FTIR spectrometer (Tensor 27, Bruker Co., Ltd., Natick, MA, USA) with a focal plane array detector (Santa Barbara Focalplane Co., Ltd., Goleta, CA, USA) and an ATR accessory (Golden Gate, Specac, Inc., Orpington, UK) placed in a macro-chamber (IMAC, Bruker Co., Ltd., Natick, MA, USA). The projected pixel sizes in the images were approximately 8 and 10 μm for the *x* and *y* directions, respectively.

Curing curves of rubber samples were acquired by the oscillatory rheological measurement using a disc curemeter (MDRH2030, M&K Co., Ltd., Chiba, Japan) at 150, 160, 170, and 180 °C. The elastic torque at each time was recorded every second to observe the curing behavior of rubber samples. The degree of cure in the curemeter $\alpha_{\mathrm{bulk}}$ was defined by:

(2) $$\alpha_{\mathrm{bulk}}\left(t\right)=\frac{M\left(t\right)-M_{\min}}{M_{\max}-M_{\min}},$$

where M is the elastic torque measured by the curemeter at each time t, $M_{\min}$ is the minimum value of the elastic torque, and $M_{\max}$ is the maximum value of the elastic torque. To characterize the viscoelastic properties of FKM and FKM/CNT, a dynamic mechanical analysis was also conducted with a rotational rheometer (ARES, TA Instruments, Inc., New Castle, DE, USA). An oscillatory shear measurement in frequency-sweep mode was conducted at 40 °C with a strain of 1%.

## 3. Results and Discussion

Figure 1b shows a series of ATR-FTIR spectra of FKM samples at different measurement times. In the spectra, two distinct absorption bands were observed: one is the C=O stretching at 1699 cm^−1^ and the other is C=C stretching band at 1656 cm^−1^ [34,39,40,41]. The evolution of the spectra was observed via the broadening of the C=O stretching band. To evaluate the spectra, the temperature profile, the integrated absorbance of the C=O stretching band, and the full width at half maximum (FWHM) were plotted against the heating time (Figure 1c–e). Both relative integrated absorbance (normalized by C–F stretching of FKM at 1396 cm^−1^) and the FWHM increased with the heating time. The change in the FWHM is attributed to the progress of the curing reaction of TAIC in FKM, which is highly correlated with the change of the crosslinking density and Young’s modulus [34]. In addition, the increase of the integrated absorbance against the heating time was observed and the increase was terminated at a certain time. Since absorbances are generally proportional to concentrations of substances, the observed increase of the integrated absorbance means the increase of the concentration of TAIC at the surface layer. The increase in the absorbance could be explained by the change of the total concentration of free TAIC and conjugated TAIC. Free TAIC is converted to conjugated TAIC (cleavage of C=C bond) caused by the curing reaction. At the surface, additional free TAIC is diffused out to the heated side, resulting in the total increase of the absorbance at the surface. The results of the diffusion of the TAIC into the surface, which has not been reported in previous work, can be elucidated by analysis of in situ ATR-FTIR spectroscopy in this study. Therefore, it can be stated that in situ ATR-FTIR spectroscopy could simultaneously observe the reaction and the diffusion of the curing agent at the surface of the rubber in the molding processes.

**Figure 2:**
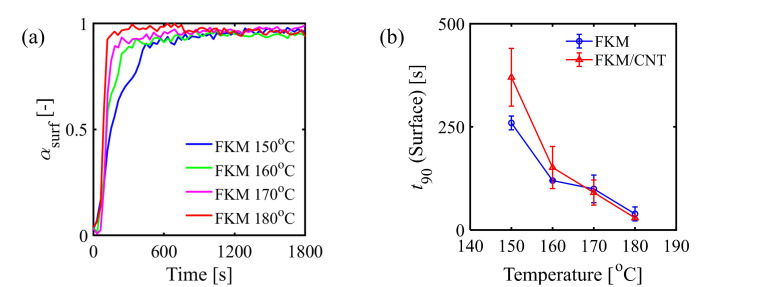
(**a**) Changes in the profiles of the reaction progress of the surface ($\alpha_{\mathrm{surf}}$, calculated by Equation (1)) measured by in situ via ATR-FTIR spectroscopy. (**b**) Comparison of the time to reach 90% of the degree of cure at the surface of FKM and FKM/CNT.

**Figure 3:**
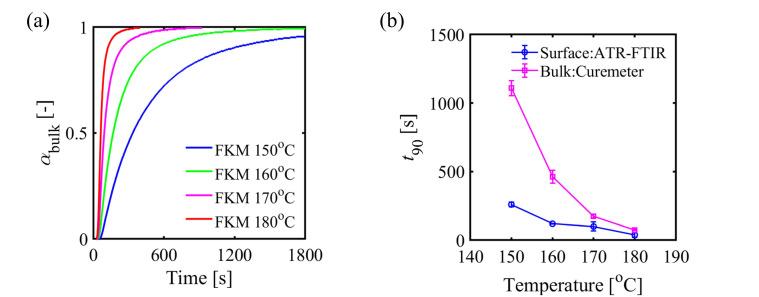
(**a**) Changes in the profiles of the reaction progress in bulk ($\alpha_{\mathrm{bulk}}$, calculated by Equation. (2)) measured by the curemeter. (**b**) Differences in the time to reach 90% of the degree of cure of FKM between the surface (in situ ATR-FTIR) and the bulk (curemeter).

To investigate the effects of the temperature and the CNT on the curing reaction rate, the time to reach 90% of the maximum achievable degree of cure ($t_{c,90}$) was calculated from the curing curves shown in Figure 2a. Figure 2b shows the temperature dependency of $t_{90}$ of both FKM and FKM/CNT samples. As the constant temperature at the heating process increased, $t_{90}$ of both samples decreased because of the acceleration of the reaction rate. The notable change here was the difference in $t_{90}$ between the FKM and FKM/CNT samples where $t_{90}$ of FKM/CNT was larger than that of FKM. This could be due to the radical trapping effect of CNTs on the retardation of the curing reaction. This retardation of curing reaction caused by the presence of CNTs was also confirmed by a kinetic study of an autocatalytic reaction model [8,14,15,16] with some curemeter data (Note S1, Figure S2, Table S1). The activation energy of the curing reaction at bulk (Table S2) was increased by the addition of CNTs. The difference of $\alpha_{\mathrm{surf}}$–heating time curves (ATR-FTIR, Figure 2a) and $\alpha_{\mathrm{bulk}}$–heating time curves (curemeter, Figure 3a) was the time needed to complete the curing reaction. Figure 3b shows $t_{90}$ of both surface and bulk of FKM samples at different heating temperatures of the heating process. $t_{90}$ of the surface was smaller than that of the bulk, and its difference was larger as the heating temperature lowered. The faster curing reaction at the surface, especially at lower temperature conditions, may be caused by the increase in concentration of TAIC at the surface, of which details are discussed in the following paragraph.

**Figure 4:**
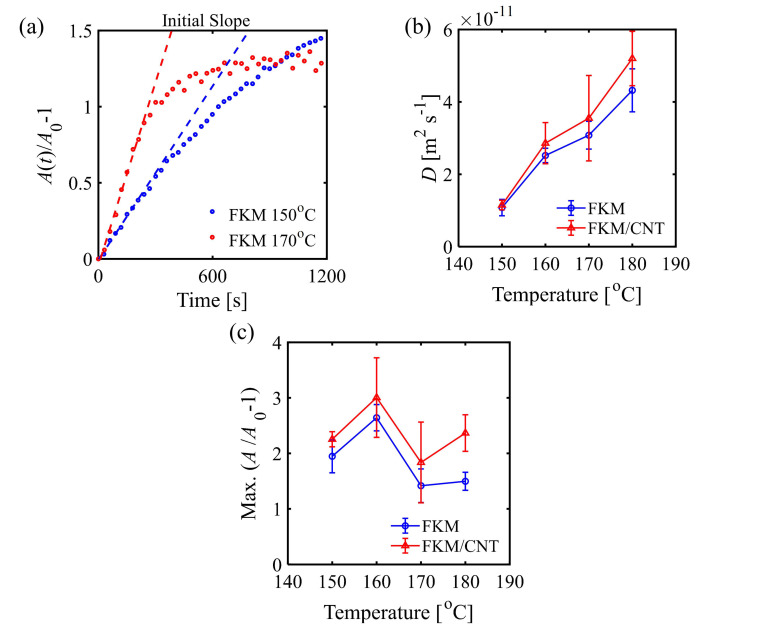
(**a**) Time-series changes in the integrated absorbance of the C=O of TAIC. (**b**) Diffusion coefficients obtained from the initial increase of the integrated absorbance. (**c**) Maximum value of the increases in the normalized integrated absorbance.

Figure 4a shows the increases in the normalized integrated absorbances of FKM during isothermal heating conditions with different heating temperatures. As the heating temperature of the heating process increased, the rate of the increase of the integrated absorbance at an initial stage after reaching the isothermal condition increased. As mentioned above, these increases of the integrated absorbance could be attributed to the diffusion of the curing agent towards the surface. To evaluate the diffusion behavior quantitatively, two-step analysis was performed: calculation of the slopes of the integrated absorbance at the beginning of the isothermal condition and conversion to the diffusion coefficient. Since the diffusion of the curing agent at the beginning of isothermal condition was less influenced by the curing reaction, the increases in the integrated absorbance could be expressed by the equation of diffusion. In this study, the Fickian diffusion model was applied to describe the diffusion behavior at the beginning of curing of the isothermal condition for estimating the diffusion coefficients (Note S2 and Figure S3). Figure 4b shows the estimated diffusion coefficients of the FKM and FKM/CNT. As the temperature increased, the diffusion rate increased. The diffusivity of the curing agent in FKM/CNT was higher than that in FKM, which was contrary to expectations. In addition, the differences in the diffusion coefficient in FKM from that in FKM/CNT became much larger, especially in high temperature conditions.

Both positive and negative effects of fillers on transport phenomena in polymeric materials [42] can be applied to the diffusivity results: restricted free volume in filler/polymer system as well as tortuous path of transfer due to obstacles of fillers can decrease the diffusion rate of a solute in polymer. On the other hand, spaces at the interfaces between the filler and polymer increase the diffusion. The diffusion rate of solute in the filler/polymer system is determined by these effects. Therefore, both dispersibility of CNT in FKM, resulting in total area of interfaces, free volume, and adhesion between CNT and FKM, affect the diffusion of the curing agent. The dispersibilities of CNT in various rubber matrices are reported in [43] from a viewpoint of solubility parameters. The solubility parameter of CNT is close to that of FKM compared with other rubber matrixes and resulted in a better improvement in electrical properties in FKM/CNT. This suggests the large area of interfaces between CNT and FKM. From a rheological viewpoint (shown in Figure S4), CNT network in FKM increases the elasticity and the viscosity, which could lead to suppression of diffusion. As for the adhesion at interfaces, there are a number of studies for chemical functionalization of CNT for further improvements of the adhesion to FKM [9,44,45]. In contrast to expectations that the dispersibility would be better and the rheological properties would be increased, the diffusivity of the curing agent in FKM/CNT measured in this study was higher than that in FKM.

Another effect of CNT is the improvement of the thermal conductivity, which may result in the difference in the temperature distribution along with the thickness direction. Since CNT possesses high thermal conductivity, the thermal conductivity of this FKM/CNT is approximately four times larger than that of FKM based on the literature [46]. The temperature distributions of FKM and FKM/CNT were calculated by the thermal diffusion equation of Fourier’s law (Note S3, Figure S5). From the calculated temperature distributions of FKM and FKM/CNT, it can be stated that the differences in temperature between FKM and FKM/CNT (induced by the thermal conductivity) were observed in the non-heated side. On the other hand, the differences in the temperatures between FKM and FKM/CNT at near-heated sides were smaller than those at the non-heated sides. Such temperature gradient at steady states of heating (Figure S6) may affect the diffusion of the curing agent.

Considering these backgrounds, possible hypotheses of factors for the diffusivity of the curing agent in the presence of CNT may be the temperature distribution in the thickness direction or insufficient interfacial adhesion (probably weakened at high temperatures) of non-functionalized CNT covering a large interfacial area. However, further future studies are required to unravel the precise mechanisms of the effects of CNT on the diffusivity of curing agents in rubber matrix. Diffusion coefficients of the curing agent measured by our method would be lowered if functionalized CNT could be introduced in FKM to increase the interfacial adhesion of CNT to FKM in the case that the interfacial adhesion largely affects the diffusion.

The observed profile of the integrated absorbance in Figure 4a also shows that the diffusion rate of the curing agent levelled out close to the completion of the curing reaction. As shown in Figure 4a, the absorbance does not always increase monotonically against the heating temperature because of the completion of the curing reaction. To compare the temperature and filler dependencies of the total amount of diffused-out curing agent, the maximum values of the normalized integrated absorbance were compared (Figure 4c). The values at 150 and 160 °C were higher than those at 170 and 180 °C because the time for diffusion to level out was more dominant than slower initial diffusion here. The overall results of in situ ATR-FTIR spectroscopy suggested that the curing temperature of 170 °C is suitable for this compound to obtain a balanced reaction rate and suppressed diffuse-out considering the competitive process of diffusion and reaction of the curing agent.

Considering both curing reaction (Figure 2) and diffusion (Figure 4), the reason for the acceleration of the curing reaction at the surface compared to that of bulk can be explained as follows. As the curing reaction proceeds, the free curing agent (TAIC) is consumed by the conjugated curing agent, which leads to the increase of the FWHM. To compensate for the loss of the free curing agent, the diffusion occurs, which leads to the increase in the total concentration of the free and conjugated curing agent. The reason there is less acceleration at higher temperatures is attributed to the faster curing reaction. This results in the fast completion of the curing reaction, which also leads to short times for the diffusion of the curing agent towards the surface. The factor determining the large acceleration of the curing reaction at lower temperature conditions can be considered a result of the longer time to complete the diffusion of the curing agent due to the slower curing reaction compared to high temperatures. The curing agent can diffuse into the surface longer so that the total amount of the curing agent is larger than that at higher temperatures, as confirmed by the maximum value of the integrated absorbance.

**Figure 5:**
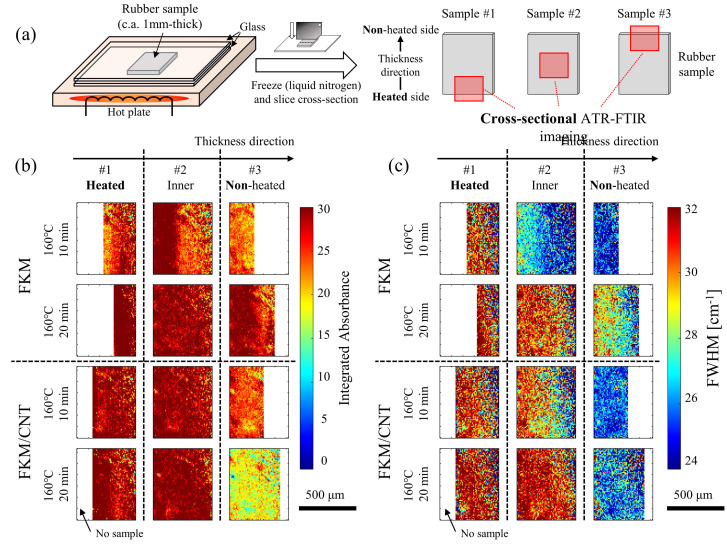
(**a**) Schematics of the cross-sectional macro ATR-FTIR spectroscopic imaging of samples heated on a hot plate. Cross-sectional spatial distribution of (**b**) the integrated absorbance and (**c**) the FWHM of FKM and FKM/CNT samples with different heating times.

To further investigate the diffusion behavior of the curing agent, based on the hypotheses of the factors for the diffusivity of the curing agent, macro ATR-FTIR spectroscopic imaging measurements were caried out. FKM and FKM/CNT samples containing the curing agent were heated on a hot plate and sliced (Figure 5a). The distributions of the curing agent and the degree of cure in the cross-sectional area of sliced samples were evaluated in the range of several hundred μm scale. Two-dimensional distributions of the integrated absorbance (Figure 5b) and the FWHM (Figure 5c) were visualized for the samples at the different heating times. Both integrated absorbance (TAIC concentration) and FWHM (degree of cure) at the non-heated side were lower than those at the heated side. The differences in the integrated absorbance between the heated and non-heated sides supported the unidirectional diffusion of the curing agent and towards the heated side this occurred during the curing process. As for the differences in the FWHM between the heated and non-heated sides, both factors affected the results, the acceleration of the curing reaction by the increased TAIC concentration at the heated surface and diffusion and the temperature distribution from the heated to the non-heated sides.

**Figure 6:**
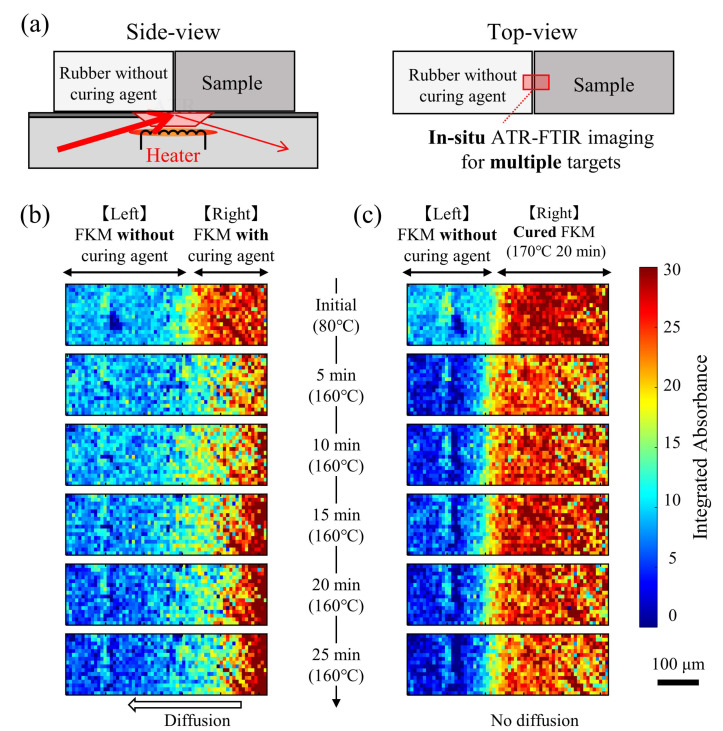
(**a**) Schematics of the in situ ATR-FTIR imaging of heating multiple targets. (**b**,**c**) Time-series changes in the two-dimensional distributions of the integrated absorbance of two specimens simultaneously measured during heating. (**b**) Differences between FKM without curing agent and FKM with curing agent, representing the diffusion of curing agent during heating. (**c**) FKM without curing agent and cured FKM, indicating no significant changes in the integrated absorbance during heating.

To investigate the diffusion behavior of the curing agent in both the non-cured and the cured samples, in situ ATR-FTIR spectroscopic imaging of heating multiple targets was conducted (Figure 6a). Two specimens, FKM with and without any curing agents were placed on the ATR crystal and the integrated absorbance at each position were visualized. Figure 6b shows in situ ATR-FTIR imaging of FKM with and without a curing agent. With the increase in the heating time, the integrated absorbance at the side of FKM with a curing agent significantly changed. The change in the integrated absorbance was owing to two factors: out-plane (thickness direction) diffusion identical to the change in Figure 4a and in-plane diffusion (perpendicular to gravity and compression direction) towards the FKM matrix without the curing agent placed on the left side. Figure 6c shows in situ ATR-FTIR imaging of cured FKM placed beside FKM without a curing agent. Compared to the results of Figure 6b, no significant difference was observed in the integrated absorbance. This indicates that the curing agent did not diffuse after the curing reaction stopped, and the diffusion is not just an effect of the gravity and compression direction. These results obtained from cross-sectional and in situ ATR-FTIR imaging are evidence of the unidirectional diffusion of the curing agent towards the heated surface and the termination of the diffusion after completion of the curing.

## 4. Conclusions

In this study, we proposed methods for characterizing the curing reaction and the diffusion behaviors of the curing agent at the surface of rubber in real time using in situ ATR-FTIR spectroscopy and spectroscopic imaging. From the investigated increases and broadenings of the C=O stretching band of the TAIC curing agent, both reaction and diffusion kinetics were quantitatively evaluated, which was difficult with the conventional method of bulk characterization through rheological measurements. The temporal changes in the FWHM of the C=O stretching band showed the reaction rate at the surface was faster than that of the bulk because the curing agent was diffused and condensed at the surface during heating.

The integrated absorbance of C=O stretching band increased with the heating time. The diffusion coefficient of the curing agent was calculated with the Fickian diffusion model from the initial slope of the integrated absorbance–heating time curve. The results showed that FKM/CNT exhibited a higher diffusivity of the curing agent and the difference in diffusion caused by the presence of CNT was much larger at higher temperatures. These were attributed to the overall effects of the temperature distribution, the adhesion of CNT and FKM, the total area of the interface, and the entanglement of the CNT network. Cross-sectional ATR-FTIR imaging results clearly visualized the unidirectional diffusion of the curing agent towards the heated surface of the FKM sample. In situ ATR-FTIR imaging also showed that the curing agent did not diffuse from the cured state, indicating the termination of the diffusion after the completion of the curing. These results are strong evidence that our proposed method is effective at elucidating the phenomenon of the kinetics of curing reactions and the diffusion of curing agents in rubber nanocomposites.

Since the proposed methods of in situ ATR-FTIR spectroscopy and spectroscopic imaging are not limited to FKM nanocomposites because the diffusion of the curing agent in the rubber matrix and restricted vibration of C=O by the reduced free volume in the rubber matrix can be applied to various rubber compounds. Therefore, we strongly believe that a wide range of rubber materials should be evaluated to characterize the kinetics at the surface of the mold. Our approach to the analysis of the curing and the diffusion behaviors of rubber nanocomposites can be useful to obtain suitable process conditions and the design of rubber compounds for exploring further high-performance products.

## Data Availability

All the data will be available to the readers.

## Supplementary Materials

The following supplemental figures, tables, and notes are available online at https://www.mdpi.com/article/10.3390/polym13172879/s1, Figure S1: Chemical structures of the materials used in this study. Figure S2: (**a**) Fitting of the curing curves measured by the curemeter. (**b**) Arrhenius plots of curing curves. Figure S3: (**a**) Calculation of initial slope of the integrated absorbance. Initial time of isothermal condition was determined from the time that FWHM started to change, indicating the beginning of curing reaction. (**b**) Relationship between the diffusion coefficient of Fickian diffusion model and the initial slope of the absorbance in this study. Figure S4: Effects of the presence of CNT in FKM on the rheological properties: the frequency-sweep of (**a**) the storage modulus and (**b**) the complex viscosity. Samples were compression-molded at 170 °C and 20 min. Figure S5: (**a**) Programmed temperatures of heating the surface to the heating temperature (170 °C). (**b**,**c**) Temperature distributions of the thickness direction in FKM and FKM/CNT calculated by unsteady thermal diffusion equation based on Fourier’s law of the boundary condition of the single-side heating. The ranges of the plots in the thickness direction are (**b**) all and (**c**) near-surface. Dotted line represents the position of the penetration depth of ATR. The chain lines are the temperature distributions of FKM and the solid lines represent the temperature distributions of FKM/CNT. Figure S6: Temperature distributions of FKM and FKM/CNT at the steady states with different heating temperature conditions. Nusselt numbers of FKM and FKM/CNT were determined to 0.100 and 0.025 based on the thermal conductivities of 0.050 and 0.020 W m^−1^ K^−1^, respectively. The heat transfer coefficient of free convection of air here used for calculation was 5 W m^−2^ K^−1^. Table S1: Parameters of the kinetic study of curing curves measured by a curemeter. Table S2: Kinetic parameters of the bulk curing reaction by the fitting of the Arrhenius plot. Note S1: Description of the kinetics analysis of the bulk curing reaction. Note S2: Description of the relationship between the increase of the absorbance and the diffusion coefficients in Fickian diffusion. Note S3: Calculation of the temperature distributions in FKM and FKM/CNT during the heating processes.
