# Supplementary material for: Novel Approaches to In-Situ ATR-FTIR Spectroscopy and Spectroscopic Imaging for Real-Time Simultaneous Monitoring Curing Reaction and Diffusion of the Curing Agent at Rubber Nanocomposite Surface
Source: Polymers (Basel). 2021 Aug 27;13(17):2879. doi: 10.3390/polym13172879 (PMC8433895; doi:10.3390/polym13172879)
Supplement: Supplementary file 1 [file polymers-13-02879-s001.zip › polymers-1355624-supplementary.pdf]

Supplementary Materials: Novel approaches of in-situ ATR-FTIR spectroscopy and spectroscopic imaging for real-time simultaneous monitoring curing reaction and diffusion of the curing agent at rubber nanocomposite surface

Shun Muroga[a]*, Yu Takahashi[b], Yuta Hikima[b]*, Seisuke Ata[a], Sergei G. Kazarian[c]*, Masahiro Ohshima[b], Toshiya Okazaki[a], Kenji Hata[a]

[a]: CNT - Application Research Center, National Institute of Advanced Industrial Science and Technology, Tsukuba Central 5, 1-1-1, Higashi, Tsukuba, Ibaraki, 305–8565, Japan

[b]: Department of Chemical Engineering, Graduate School of Engineering, Kyoto University, Nishikyo-ku, Kyoto, 615–8510, Japan

[c]: Department of Chemical Engineering, Imperial College London, South Kensington Campus, London, SW7 2AZ, United Kingdom

*Correspondence: to S. Muroga (muroga-sh@aist.go.jp), Y. Hikima (hikima@cheme.kyoto-u.ac.jp), S. G. Kazarian (s.kazarian@imperial.ac.uk)

Table of Contents

List of Figures

Figure S1. Chemical structures of the materials used in this study. 4

Figure S2. (a) Fitting and (b) Arrhenius plot of the curing curves measured by the curemeter. 5

Figure S3. (a) Calculation of initial slope of the integrated absorbance (identical to **Figure 4** (a)). (b) Relationship between the diffusion coefficient of Fickian diffusion model and the initial slope of the absorbance in this study. 10

Figure S4. Effects of the presence of CNT in FKM on the rheological properties: the frequency-sweep of (a) the storage modulus and (b) the complex viscosity. Samples were compression-molded at 170°C and 20 min. 11

Figure S5. (a) Programmed temperatures of heating the surface to the isothermal temperature (170°C). (b,c) Temperature distributions of the thickness direction in FKM and FKM/CNT calculated by unsteady thermal diffusion equation based on Fourier's law of the boundary condition of the single-side heating. The ranges of the plots in the thickness direction are (b) all and (c) near-surface. Dotted line represents the position of the penetration depth of ATR. The chain lines are the temperature distributions of FKM, and the solid lines represent the temperature distributions of FKM/CNT........................... 14

Figure S6. Temperature distributions of FKM and FKM/CNT at the steady states with different isothermal temperature conditions. Nusselt numbers of FKM and FKM/CNT were determined to 0.100 and 0.025 based on the thermal conductivities of 0.050 and 0.020 W m^{-1} K^{-1}, respectively. The heat transfer coefficient of free convection of air here used for calculation was 5 W m^{-2} K^{-1}.. 15

List of Tables

Table S1. Parameters of the kinetic study of curing curves measured by a curemeter.......... 5

Table S2. Kinetic parameters of the bulk curing reaction by the fitting of the Arrhenius plot. 6

List of Notes

Note S1. Description of the kinetics analysis of the bulk curing reaction. 4

Note S2. Description of the relationship between the increase of the absorbance and the diffusion coefficients in Fickian diffusion. 6

Note S3. Calculation of the temperature distributions in FKM and FKM/CNT during the heating processes. 11

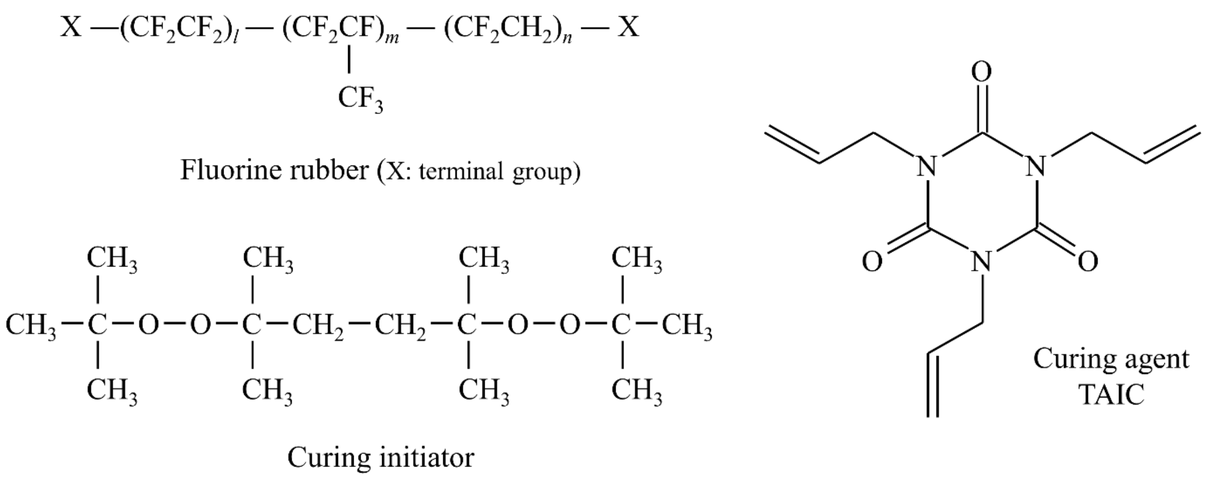

Figure S1. Chemical structures of the materials used in this study.

Note S1. Description of the kinetics analysis of the bulk curing reaction.

The kinetics analysis of the bulk curing reaction was conducted based on the following equations of the autocatalytic reaction model [8,14-16].

$$\frac{\mathrm{d}\alpha_{\mathrm{bulk}}}{\mathrm{d}t} = k\alpha_{\mathrm{bulk}}^{m}(1-\alpha_{\mathrm{bulk}})^{n} \quad (1)$$

$$k = k_0 \exp\left(-\frac{E_{\mathrm{a}}}{RT}\right) \quad (2)$$

where m and n are the orders of reaction, E_{a} is the activation energy, k_0 is a pre-exponential factor, and R is the universal gas constant. The curves of the α_{bulk} were smoothed by Savitzky-Golay filtering with a polynomial order of three for the calculation of the first derivative of the degree of cure, $\mathrm{d}\alpha_{\mathrm{bulk}}/\mathrm{d}t$. Fitting of the profile of $\mathrm{d}\alpha_{\mathrm{bulk}}/\mathrm{d}t$ against α_{bulk} (**Figure S2**(a)) was conducted in the range of $\alpha_{\mathrm{bulk}} \leq 0.8$ because the derivative in the range of higher degree of cure was fluctuated due to the derivative of small increase at the last stage of curing reaction. After the fitting shown in **Figure S2**(a), the three parameters, m, n, k, were determined (listed in **Table S1**).

Then, from the Arrhenius plots (linear approximation between $\ln(k)$ vs $1/T$, shown in **Figure S2**(b)), the activation energy and the pre-exponential factor were obtained (**Table S2**).

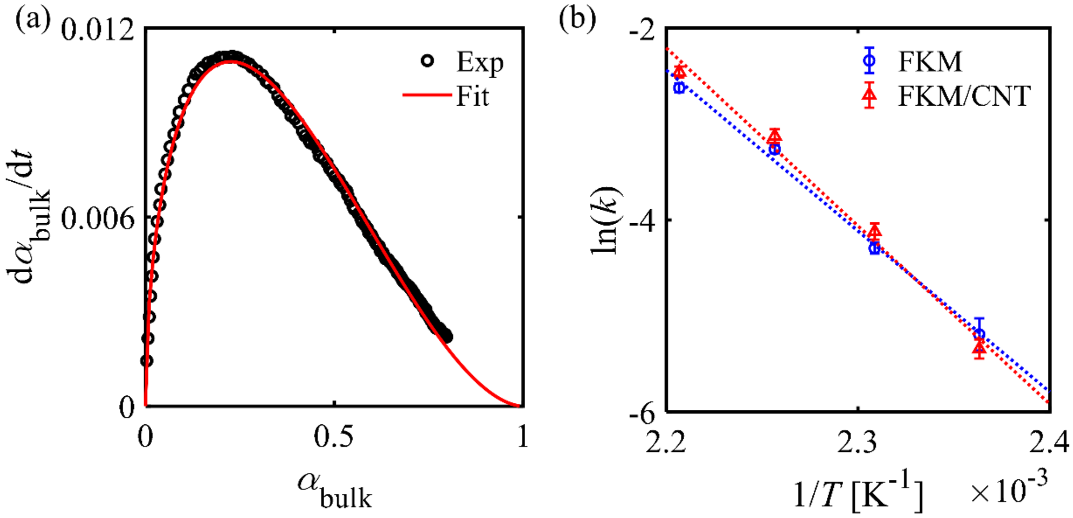

Figure S2. (a) Fitting and (b) Arrhenius plot of the curing curves measured by the curemeter.

Table S1. Parameters of the kinetic study of curing curves measured by a curemeter.

Material	*T* [°C]	1/*T* [1/K]	ln*k*	*m*	*n*	*m*+*n*
FKM	150	0.00236	-5.193	0.301	1.89	2.191
FKM	160	0.00231	-4.294	0.365	1.826	2.191
FKM	170	0.00226	-3.263	0.522	1.767	2.288
FKM	180	0.00221	-2.624	0.59	1.464	2.055
FKM/CNT	150	0.00236	-5.345	0.299	1.627	1.926
FKM/CNT	160	0.00231	-4.121	0.397	1.665	2.062
FKM/CNT	170	0.00226	-3.126	0.509	1.638	2.146
FKM/CNT	180	0.00221	-2.459	0.548	1.217	1.765

Table S2. Kinetic parameters of the bulk curing reaction by the fitting of the Arrhenius plot.

	E_a [kJ/mol]	$\ln k_0$	R^2
FKM	127.95	31.4	0.994
FKM/CNT	154.28	38.6	0.988

Note S2. Description of the relationship between the increase of the absorbance and the diffusion coefficients in Fickian diffusion.

The Fickian diffusion was governed by the following equation:

$$\frac{\partial C(t,z)}{\partial t} = D\frac{\partial^2 C(t,z)}{\partial z^2} \quad (3)$$

Where D is the diffusion coefficients [$m^2 s^{-1}$], t is the time [s], and z is the coordinate of the thickness direction of the sample [m]. The boundary conditions of the model are described under the assumption of the uniform concentration of the component (C_0) in the sample having the thickness of L:

$$C = C_0 \ (x < L) \text{ at } t = 0 \quad (4)$$

$$C = 0 (x > L) \text{ at } t = 0 \quad (5)$$

From Eqs. (3)-(5), the concentration was determined to

$$C(t,z) = \frac{C_0}{2}\left\{\text{erf}\left(\frac{L-z}{2\sqrt{Dt}}\right) + \text{erf}\left(\frac{L+z}{2\sqrt{Dt}}\right)\right\} \quad (6)$$

where erf is the Gauss's error function defined as

$$\text{erf}(x) = \int_0^x \exp(-\xi^2)\,\text{d}\xi \quad (7)$$

The total amount of the diffused-out component $M(t)$ was calculated by

$$M(t) = C_0L - \int_0^L C(t,z)\mathrm{d}z \quad (8)$$

In case of this study, ATR-FTIR detects the amount of both diffused-out component and remaining component within the depth that evanescent wave penetrates. Therefore, the increase of the absorbance by diffusion during heating can be modelled by the following equation.

$$\begin{aligned}\Delta A(t) &\propto M(t) + \int_{L-\Delta z}^{L} C(t,z)\mathrm{d}z \\ &= C_0L - \int_0^{L-\Delta z} C(t,z)\mathrm{d}z\end{aligned} \quad (9)$$

Where Δz is the depth that the evanescent wave penetrates. In this study, sample thickness L is 1 mm (1×10^{-3} m), and Δz is represented here by the penetration depth of 0.93 μm (9.3×10^{-7} m) to analyze data. Substituting Eq. (6) into Eq. (9) yields

$$\begin{aligned}\Delta A(t) &\propto C_0L - \frac{C_0}{2}\left\{\int_0^{L-\Delta z} \mathrm{erf}\left(\frac{L-z}{2\sqrt{Dt}}\right)\mathrm{d}z \right. \\ &\left. + \int_0^{L-\Delta z} \mathrm{erf}\left(\frac{L+z}{2\sqrt{Dt}}\right)\mathrm{d}z\right\} \\ &= C_0L - \frac{C_0}{2}\left(f_1(t) + f_2(t)\right)\end{aligned} \quad (10)$$

Here, integrals of the Gauss's error function were tentatively defined as $f_1(t)$ and $f_2(t)$. The integral of the Gauss's error function is not easily solved due to its complexity. Here, following Taylor's expansion of the Gauss's error function is introduced:

$$\mathrm{erf}(x) = \frac{2}{\sqrt{\pi}}\sum_{n=0}^{\infty}\left(\frac{(-1)^n x^{2n+1}}{n!\,(2n+1)}\right) \quad (11)$$

The integral of the Gauss's error function is subsequently described as:

$$\int \mathrm{erf}(x)\,dx = \frac{2}{\sqrt{\pi}} \sum_{n=0}^{\infty} \left(\frac{(-1)^n x^{2n+2}}{2(n+1)!\,(2n+1)} \right) + \text{const.} \tag{12}$$

Thus, the integrals of the Gauss's error function in Eq. (10) are expressed by the following equations:

$$\begin{aligned} f_1(t) &= \int_0^{L-\Delta z} \mathrm{erf}\left(\frac{L-z}{2\sqrt{Dt}}\right) \mathrm{d}z \\ &= 2\sqrt{Dt} \int_{\frac{\Delta z}{2\sqrt{Dt}}}^{\frac{L}{2\sqrt{Dt}}} \mathrm{erf}(z')\,\mathrm{d}z' \quad \left(\text{Notice: } z' = \frac{L-z}{2\sqrt{Dt}}\right) \\ &= 2\sqrt{Dt}\frac{2}{\sqrt{\pi}} \sum_{n=0}^{\infty} \left(\frac{(-1)^n}{2(n+1)!\,(2n+1)} \left\{ \left(\frac{L}{2\sqrt{Dt}}\right)^{2n+2} \right.\right. \\ &\quad \left.\left. - \left(\frac{\Delta z}{2\sqrt{Dt}}\right)^{2n+2} \right\} \right) \end{aligned} \tag{13}$$

$$\begin{aligned} f_2(t) &= \int_0^{L-\Delta z} \mathrm{erf}\left(\frac{L+z}{2\sqrt{Dt}}\right) \mathrm{d}z \\ &= 2\sqrt{Dt} \int_{\frac{L}{2\sqrt{Dt}}}^{\frac{2L-\Delta z}{2\sqrt{Dt}}} \mathrm{erf}(z")\,\mathrm{d}z" \quad \left(\text{Notice: } z" = \frac{L+z}{2\sqrt{Dt}}\right) \\ &= 2\sqrt{Dt}\frac{2}{\sqrt{\pi}} \sum_{n=0}^{\infty} \left(\frac{(-1)^n}{2(n+1)!\,(2n+1)} \left\{ \left(\frac{2L-\Delta z}{2\sqrt{Dt}}\right)^{2n+2} \right.\right. \\ &\quad \left.\left. - \left(\frac{L}{2\sqrt{Dt}}\right)^{2n+2} \right\} \right) \end{aligned} \tag{14}$$

Therefore, the increase of the absorbance is described by:

$$
\begin{aligned}
\Delta A &\propto C_0 L - \frac{C_0}{2}\left\{\int_0^{L-\Delta z} \mathrm{erf}\left(\frac{L-z}{2\sqrt{Dt}}\right) \mathrm{d}z + \int_0^{L-\Delta z} \mathrm{erf}\left(\frac{L+z}{2\sqrt{Dt}}\right) \mathrm{d}z\right\} \\
&= C_0 L - \frac{C_0}{2}\left(f_1(t) + f_2(t)\right) \\
&= C_0 L \\
&- \frac{C_0}{2}\left(2\sqrt{Dt}\frac{2}{\sqrt{\pi}}\sum_{n=0}^{\infty}\left(\frac{(-1)^n}{2(n+1)!\,(2n+1)}\left\{\left(\frac{2L-\Delta z}{2\sqrt{Dt}}\right)^{2n+2}\right.\right.\right. \\
&\left.\left.\left.- \left(\frac{\Delta z}{2\sqrt{Dt}}\right)^{2n+2}\right\}\right)\right) \qquad (15) \\
&= C_0 L\left(1\right. \\
&- \frac{1}{L\sqrt{\pi}}\sum_{n=0}^{\infty}\left(\frac{(-1)^n 2^{-(2n+1)}}{(n+1)!\,(2n+1)}\{(2L-\Delta z)^{2n+2}\right. \\
&\left.\left.- (\Delta z)^{2n+2}\}D^{-\frac{2n+1}{2}}t^{-\frac{2n+1}{2}}\right)\right)
\end{aligned}
$$

Based on the Lambert-Beer law, the absorbance is proportional to the concentration of the component. The absorbance at the beginning can be expressed by

$$A(0) = \epsilon C_0 \Delta z \text{ (Notice: } \epsilon\text{: const.)} \qquad (16)$$

Finally, the ratio of the increase of the absorbance to the initial absorbance can be describes as following equation:

$$\frac{\Delta A}{A_0} = \frac{A}{A_0} - 1$$

$$= \frac{1}{\Delta z/L}\left(1 - \frac{1}{L\sqrt{\pi}}\sum_{n=0}^{\infty}\left(\frac{(-1)^n 2^{-(2n+1)}}{(n+1)!\,(2n+1)}\{(2L-\Delta z)^{2n+2} - (\Delta z)^{2n+2}\}D^{-\frac{2n+1}{2}}t^{-\frac{2n+1}{2}}\right)\right) \quad (17)$$

By fitting the beginning of the profile of this equation, the relationship between the diffusion coefficient [m^2/s] and the initial slope of the normalized absorbance (i.e., how many times the absorbance increases per second in **Figure S3**(a)) was obtained as shown in **Figure S3** (b).

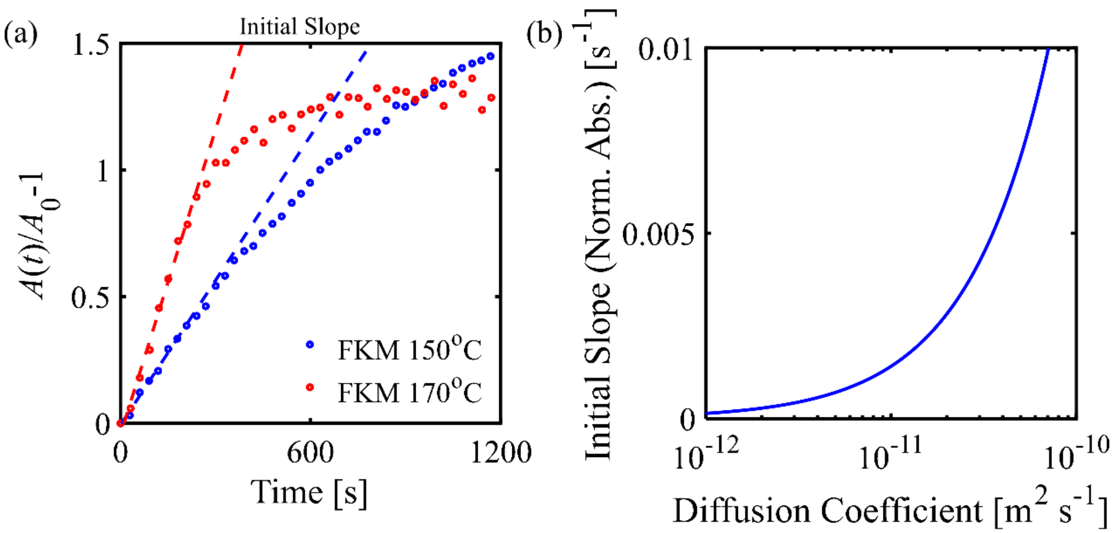

Figure S3. (a) Calculation of initial slope of the integrated absorbance (identical to **Figure 4** (a)). (b) Relationship between the diffusion coefficient of Fickian diffusion model and the initial slope of the absorbance in this study.

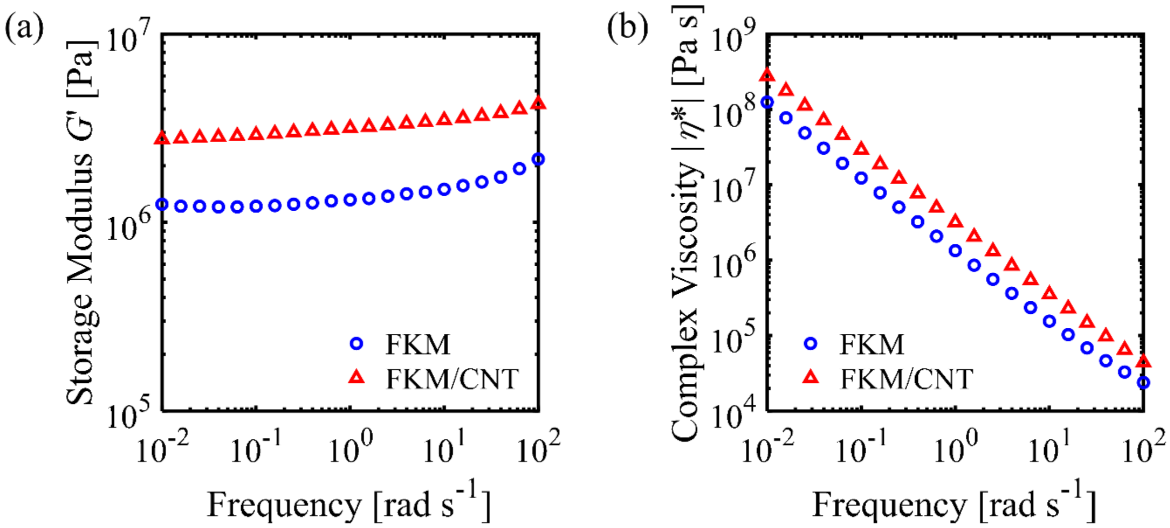

Figure S4. Effects of the presence of CNT in FKM on the rheological properties: the frequency-sweep of (a) the storage modulus and (b) the complex viscosity. Samples were compression-molded at 170°C and 20 min.

Note S3. Calculation of the temperature distributions in FKM and FKM/CNT during the heating processes.

Unidirectional thermal conduction of the thickness direction in a sample can be described by unsteady thermal diffusion equation based on Fourier's law:

$$\frac{\partial T_s(t,z)}{\partial t} = \frac{\partial}{\partial z}\left(\frac{k}{\rho C_p}\frac{\partial T_s(t,z)}{\partial z}\right) = \alpha \frac{\partial^2 T_s(t,z)}{\partial z^2} \tag{18}$$

where T_s is the temperature of the sample [°C], t is the time [s], z is the coordinate of the thickness direction [m], α is the thermal diffusivity [m^2 s^{-1}], k is the thermal conductivity [W m^{-1} K^{-1}], ρ is the density [kg m^{-3}], and C_p is the specific heat capacity [J kg^{-1} K^{-1}]. $\rho =$

$1900\ \mathrm{kg\ m^{-3}}$ and $C_p = 2000\ \mathrm{J\ kg^{-1}\ K^{-1}}$ were used as typical values of FKM. As for thermal conductivity, approximates of 0.050 W m^{-1} K^{-1} (FKM) and 0.020 W m^{-1} K^{-1} (FKM/CNT 1.0 phr) were used based on the literature [46] with an assumption of the sufficiently small effect of the degree of cure on the thermal conductivity and the exothermal enthalpy generated by the curing reaction. The boundary conditions are given as:

$$T_{\mathrm{s}}(t, z = 0) = T_{\mathrm{s,H}}(t) \tag{19}$$

$$T_{\mathrm{s}}(t, z = L) = T_{\mathrm{s,L}}(t) \tag{20}$$

where L is the sample thickness [m], $T_{\mathrm{s,H}}$ and $T_{\mathrm{s,L}}$ are the temperatures of the sample at the heated and non-heated sides. Initial condition of the temperature in the sample is as follows:

$$T_{\mathrm{s}}(t = 0, 0 \leq z \leq L) = T_{\mathrm{RT}} \tag{21}$$

where T_{RT} is the atmospheric temperature at the initial conditions, i.e. room temperature (30°C). As for heated side, considering the thermal conductions is sufficiently high (i.e., sufficiently large heat transfer coefficient due to the sufficient contact between ATR crystal and the sample), the temperature of the surface of the heated side is equivalent to the programmed temperature of heating the surface T_{H}.

$$T_{\mathrm{s,H}}(t) = T_{\mathrm{H}}(t) \tag{22}$$

The programmed temperature of the heating is expressed by the following equations:

$$T_{\mathrm{H}}\left(t < \frac{T_{\mathrm{iso}} - T_{\mathrm{RT}}}{\beta}\right) = T_{\mathrm{RT}} + \beta t \tag{23}$$

$$T_{\mathrm{H}}\left(t \geq \frac{T_{\mathrm{iso}} - T_{\mathrm{RT}}}{\beta}\right) = T_{\mathrm{iso}} \tag{24}$$

where β is the heating rate (20°C min^{-1} = 1/3 °C s^{-1} in this study), and T_{iso} is the isothermal temperature of the heating process (150, 160, 170, and 180°C). In the thermal conduction in the non-heated surface, the heat transfer cooled by the atmosphere (30°C) is expressed as follows:

$$q_{\mathrm{L}}(t) = h_{\mathrm{L}}(T_{\mathrm{s,L}}(t) - T_{\mathrm{RT}}) \tag{25}$$

where q_{L} is the heat cooled by the atmosphere at the non-heated surface, h_{L} is the heat transfer coefficient [W m^{-2} K^{-1}]. Heat balance at the non-heated side ($z = L$) is then expressed as follows:

$$\frac{\partial T_{\mathrm{s}}(t, z = L)}{\partial t} = \frac{\partial}{\partial z}\left(\alpha \frac{\partial T_{\mathrm{s}}(t, z = L)}{\partial z} - \frac{q_{\mathrm{L}}(t)}{\rho C_p}\right) \tag{26}$$

Calculations of the thermal diffusion equation were conducted in the time intervals of 1.0×10^{-4} s. The acquired times-series changes in the temperature distributions are shown in **Figure S5**. In the case of the steady state (sufficient time and $T_{\mathrm{H}} = T_{\mathrm{iso}}$), the temperature at the non-heated surface can be expressed by the heat balances:

$$T_{\mathrm{s}}(z = L) = \frac{(hL/k)T_{\mathrm{RT}} + T_{\mathrm{iso}}}{1 + hL/k} = \frac{NuT_{\mathrm{RT}} + T_{\mathrm{iso}}}{1 + Nu} \tag{27}$$

where Nu is the dimensionless value of Nusselt number representing the ratio of convective to conductive heat transfer at the interface ($Nu = hL/k$). The temperature distribution at the steady state can be described as follows:

$$\frac{T_{\mathrm{iso}} - T(z)}{T_{\mathrm{iso}} - T_{\mathrm{RT}}} = \frac{z}{L}\frac{Nu}{1 + Nu} \tag{28}$$

In the present analysis, $h_{\mathrm{L}} = 5\ \mathrm{W\ m^{-2}\ K^{-1}}$ for the typical value of the heat transfer coefficient of free convection of air was supposed and the Nusselt numbers of 0.100 and 0.025 for FKM and

FKM/CNT were used to calculate the time-series changes in the temperature distributions in the sample. Calculated temperature distributions at the steady states with different isothermal temperatures are shown in **Figure S6**.

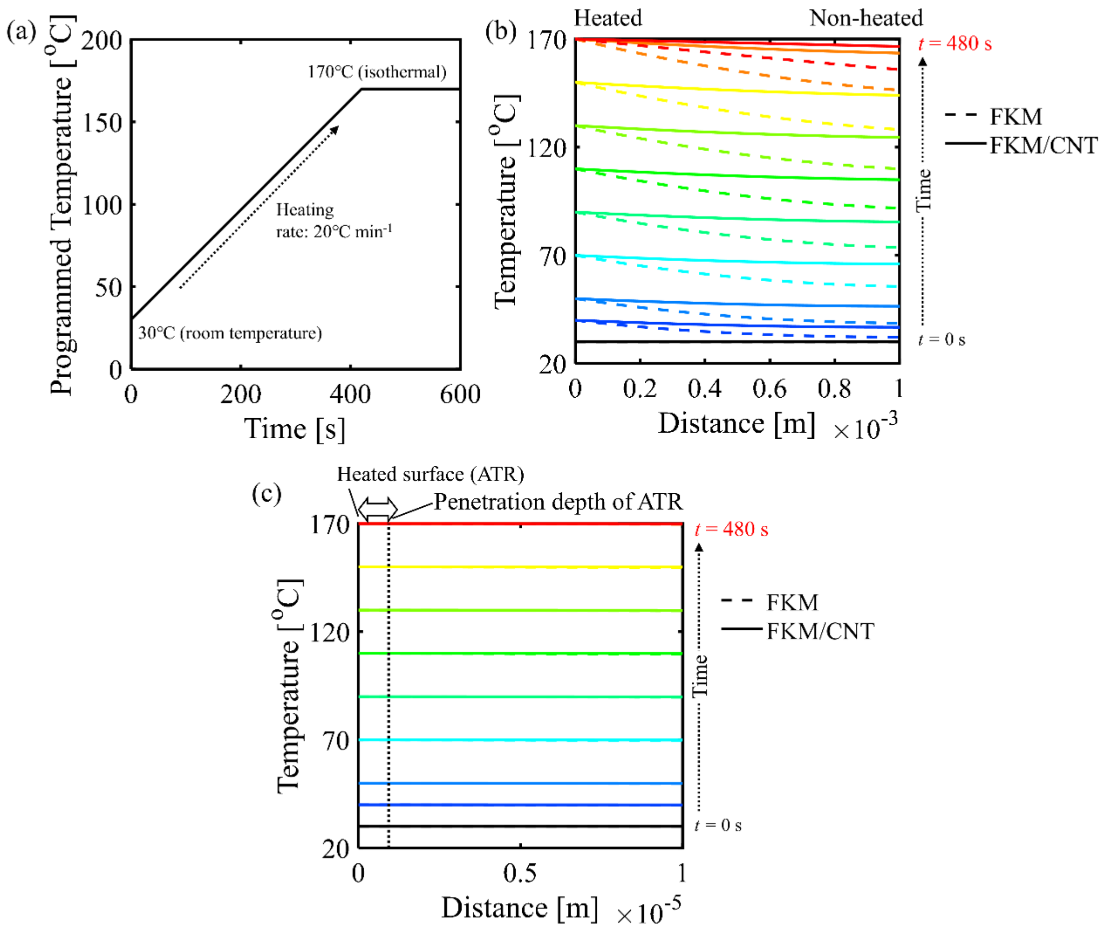

Figure S5. (a) Programmed temperatures of heating the surface to the isothermal temperature (170°C). (b,c) Temperature distributions of the thickness direction in FKM and FKM/CNT calculated by unsteady thermal diffusion equation based on Fourier's law of the boundary condition of the single-side heating. The ranges of the plots in the thickness direction are (b) all and (c) near-surface. Dotted line represents the position of the penetration depth of ATR. The chain lines are the temperature distributions of FKM, and the solid lines represent the temperature distributions of FKM/CNT.

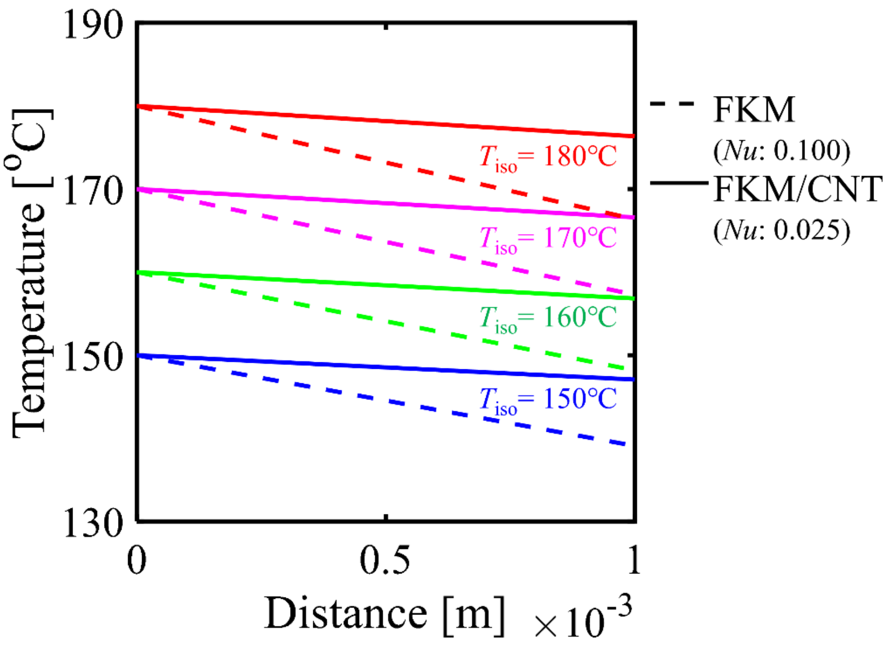

Figure S6. Temperature distributions of FKM and FKM/CNT at the steady states with different isothermal temperature conditions. Nusselt numbers of FKM and FKM/CNT were determined to 0.100 and 0.025 based on the thermal conductivities of 0.050 and 0.020 W m^{-1} K^{-1}, respectively. The heat transfer coefficient of free convection of air here used for calculation was 5 W m^{-2} K^{-1}.
